# Study of a Narrow Fabric-Based E-Textile System—From Research to Field Tests

**DOI:** 10.3390/s24144624

**Published:** 2024-07-17

**Authors:** Paula Veske-Lepp, Bjorn Vandecasteele, Filip Thielemans, Vera De Glas, Severine Delaplace, Bart Allaert, Kurt Dewulf, Annick Depré, Frederick Bossuyt

**Affiliations:** 1 Centre for Microsystems Technology (CMST), Interuniversity Microelectronics Centre (IMEC), Ghent University, Technologiepark 126, 9052 Gent, Belgium; bjorn.vandecasteele@ugent.be (B.V.); filip.thielemans@ugent.be (F.T.); 2 Apparel Division, Group SIOEN Industries, SIOEN NV, Fabriekstraat 23, 8850 Ardooie, Belgium; 3 Center of Technology Connect Group (CTC), Bargiestraat 2, 8900 Ieper, Belgium; 4 Elasta Ind., Textielstraat 15, 8790 Waregem, Belgium

**Keywords:** e-textiles, wearables, durability, wearability, user-acceptance

## Abstract

Electronic textiles (e-textiles) are a branch of wearable technology based on integrating smart systems into textile materials creating different possibilities, transforming industries, and improving individuals’ quality of life. E-textiles hold vast potential, particularly for use in personal protective equipment (PPE) by embedding sensors and smart technologies into garments, thus significantly enhancing safety and performance. Although this branch of research has been active for several decades now, only a few products have made it to the market. Achieving durability, reliability, user acceptance, sustainability, and integration into current manufacturing processes remains challenging. High levels of reliability and user acceptance are critical for technical textiles, such as those used in PPE. While studies address washing reliability and field tests, they often overlook end user preferences regarding smart textiles. This paper presents a narrow fabric-based e-textile system co-developed by engineers, garment and textiles’ manufacturers, and firefighters. It highlights material choices and integration methods, and evaluates the system’s reliability, sustainability, and user experience, providing comprehensive insights into developing and analyzing e-textile products, particularly in the PPE field.

## 1. Introduction

E-textiles are changing wearable technology by allowing electronics to be seamlessly integrated into fabrics, apparel, and other textile products. Integrating electronics into textiles opens up several possibilities, changing industries and enhancing the quality of life of individuals. The potential applications of e-textiles are vast and diverse. One of the fields e-textiles can support is personal protective equipment (PPE) through the incorporation of sensors and smart technologies into garments [1,2]. These sensors and systems can provide real-time monitoring, e.g., of environmental conditions, for enhanced safety and performance [3,4]. This innovation has the potential to greatly improve the protection and well-being of individuals working in hazardous environments.

There are several challenges in the PPE field, such as the risk of sustaining burn wounds during interventions [5,6,7], overexertion [8], and hyperthermia [9]. One example, the PROeTEX project, focused on developing a smart t-shirt system to enable the detection of users’ health parameters, such as heart rate and posture, and environmental variables, like external temperature, and heat flux passing through the garments [10,11]. Moreover, Soukupand Blecha (2014) [7] developed a firefighter protective suit that is capable of monitoring heart rate (HR), monitoring users’ movements, detecting toxic gasses in the environment, and measuring temperature and relative humidity inside and outside of the suit.

However, achieving the required durability, reliability, user acceptance, and sustainability and integrating the system production into current (textile or clothing) manufacturing processes are inhibiting applications from reaching the market [12,13,14]. High rates of reliability (no failures due to wearing, washing, etc.) and user acceptance are highly important for technical textiles used in applications such as PPE [15,16]. Even though some studies mention washing reliability and even field tests, the data do not capture the views and preferences of end users regarding smart textiles [7,11]. Moreover, the complexity of seamlessly integrating electronic components into fabrics without compromising their inherent properties, such as flexibility and comfort, remains a significant challenge [17]. For example, soldering, conductive adhesives, and snap connectors offer varying degrees of success, but each has limitations in terms of mechanical robustness and ease of integration.

The issue of (environmental) sustainability must not be overlooked either. The concept of environmental sustainability encompasses a number of aspects, such as durability, recycling/reuse/disassembly possibilities, or material and design choices [18,19]. For example, product durability can extend product lifecycles, resulting in fewer products being needed [20]. However, is it still worth it if the materials are not recyclable or reusable? Without recyclable, reusable, and efficient (sourcing of) materials, the long-lasting nature of a product may be overshadowed by the negative impact it has on the environment when it eventually reaches the end of its life cycle. Also, it is important to carefully consider the design of the e-textile system and product [21,22,23]. For example, products with modular parts can be easily disassembled and replaced, instead of being discarded as a whole.

**Figure 1:**
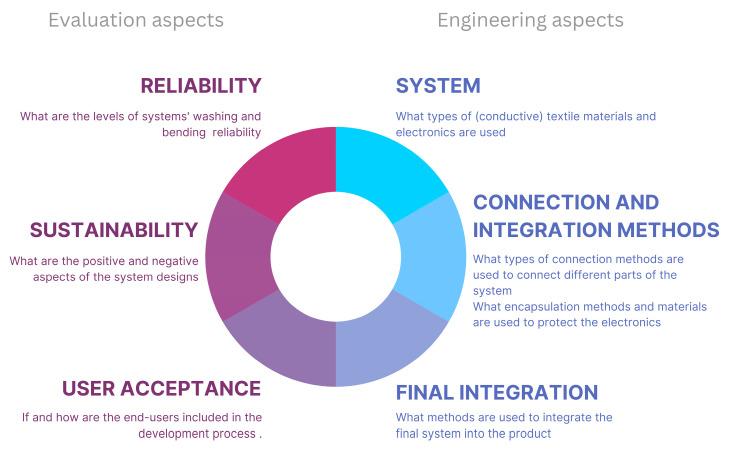
Overview of aspects (engineering and evaluation) covered in this work to develop and analyze an e-textile product. The figure shows a closed loop of 6 aspects that are divided into two halves.

To address these challenges, this paper covers a combination of research, practical experimentation, and field tests executed by firefighters. The article demonstrates a narrow fabric-based e-textile system co-developed by electronics, textile, and apparel engineers together with end users (firefighters). The co-development included first specifying the needs and habits (protocols) of the end users and then designing the e-textile system based on that. Each round of prototype development and testing always included the end users but was also always checked for safety and basic reliability, such as washing and mechanical stress. Moreover, the environmental sustainability aspect is also considered as an important factor and is analyzed. Thus, this paper aims to highlight the choices made during the development process, such as choosing narrow fabrics to make and integrate a full smart system into PPE. The study also highlights both fixed (soldered connection) and detachable (use of magnets and pogo pins) connection methods for the system [17]. Figure 1 shows an overview of aspects covered to develop and analyze an e-textile product, specifically one intended for use in the PPE field.

This work addresses two larger aspects of the e-textile system: (1) engineering/development and (2) evaluation. The materials and methods used to make choices about the system are introduced in five sections: (1) electronics; (2) textiles; (3) the electronics’ integration; (4) the system parts connection; and (5) the final system integration methods. Moreover, reliability, sustainability (easy repair and/or reusing parts), and user experience are reviewed and discussed, providing solutions to the key challenges faced in advancing PPE e-textiles towards market readiness.

## 2. Materials and Methods

**Figure 2:**
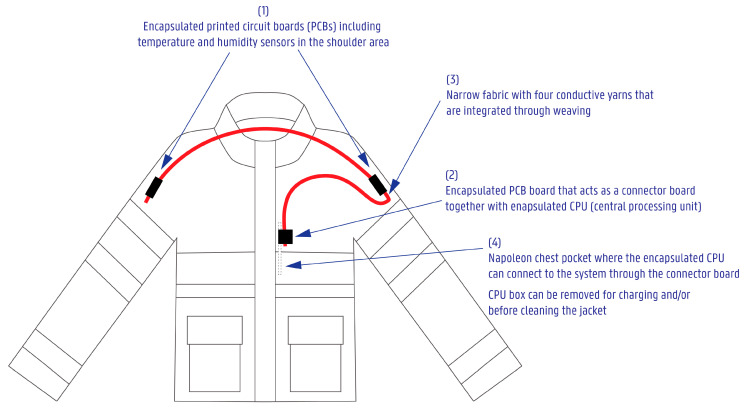
Simplified technical drawing of the firefighter jacket with the smart system.

Figure 2 provides an overview of the full system and how it is integrated into the firefighter jacket.

The Materials and Methods section is written in five sections to highlight the development of and materials used for each part of the total e-textile system. The five sections are as follows:

1. Electronics: this section introduces the electronics used in the e-textile system.
2. Textiles: this section introduces the textile parts—narrow fabrics and conductive yarns—used to fabricate e-textile system.
3. Materials and methods used for the electronics’ integration: this section introduces all the materials and methods used to encapsulate, coat and integrate electronics to make a reliable e-textile system.
4. Materials and methods used for the different system parts’ connection: this section introduces all the materials and methods used to connect different parts of the e-textile system to each other, such as battery and central processing unit (CPU) to the sensors.
5. Methods used for the final system’s integration into the product: this section introduces the method used to integrate the full e-textile system to the final end-user product (firefighter jacket) and summarizes the whole Materials and Methods section

### 2.1. Electronics

The electronics within the narrow fabrics include the following (Figure 2 parts 1 and 2):

1. Two printed circuit boards (PCBs) including a temperature and humidity sensor.
2. One PCB board designed to act as a connector board to connect the narrow fabric with the external CPU board and box. CPU also included an auditory alarm that started working after reaching a certain temperature level.

**Figure 3:**
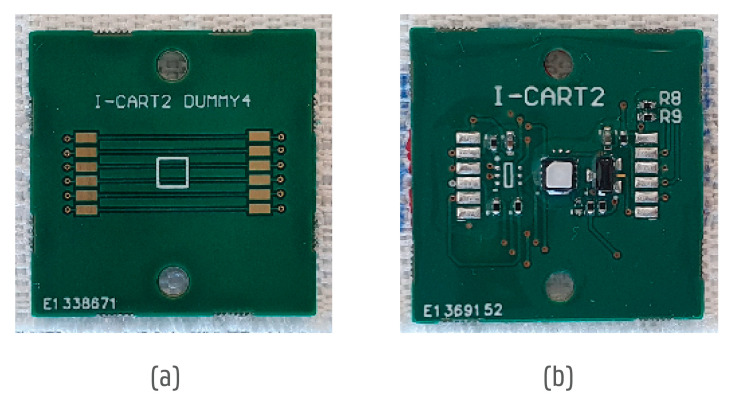
Overview of sensor boards used in testing: (**a**) non-functional board to test general reliability tests for textile and encapsulation materials; (**b**) functional final sensor board. The boards were of the same size (25 × 25 mm), shape, and thickness (1 mm).

The PCBs were coated with ELPEGUARD^®^ Twin-Cure^®^ DSL 1600 E-FLZ (Lackwerke Peters GmbH & Co. KG, Kempen, Germany) to accommodate high humidity and mechanical strains. While the initial integration tests were performed using non-functional (no assembled components) boards, later on, functional boards were used (Figure 3). The connector board was 30 × 15 mm in size and had connection pads to connect to the yarns in the narrow fabric to the CPU box separately.

### 2.2. Textiles

Polyester-based narrow fabrics integrated with six conductive yarns were used for interconnections to connect the electronics and carry low-power and data signals. The narrow fabric was polyester-based as it was integrated into the jacket between the layers. Laboratory tests proved that the use of an aramid-based ribbon was not necessary as the temperature between the jacket layers would not reach high enough temperature to melt the fabric [24]. 

**Figure 4:**
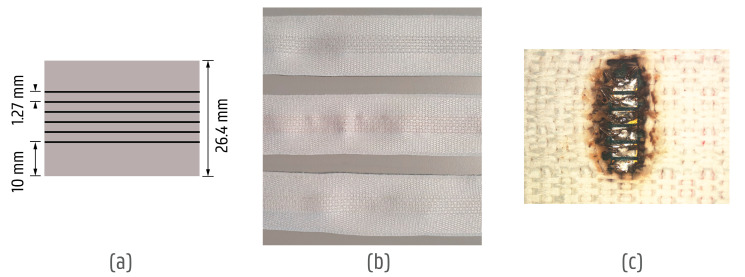
Overview of yarn integrations in the narrow fabric. (**a**) Narrow fabric dimensions (width 26.4 mm) with the yarn pitch (1.27 mm). (**b**) Comparison of 3 yarn integrations, Yarn 5, Yarn 1, and Yarn 2, based on the descriptions in Table 1. (**c**) Laser-cut opening in the narrow fabric to expose the yarns locally and solder them to the PCB.

The pitch between the yarns was set to 1.27 mm and the narrow fabric width was 26.4 mm (Figure 4a). The conductive yarns were integrated into the middle area, so the edges of the fabric could be used for sewing the system in the product (Figure 4b).

Seven different conductive yarns were tested for reliability and ease of integration (Table 1). The requirement was that soldering to the yarn had to be possible. Thus, stainless steel (plated) yarns could not be used but using steel-based yarns with copper plating was possible. Based on the knowledge gathered from the literature and previous work [25,26,27,28], the yarn also had to have a coating for additional protection from washing, sweat, and wear and tear. The yarn had to withstand a minimum of 25 washing cycles at 60 degrees Celsius and type 3 detergent according to the ISO 6330-2012 standard [29]. Considering that the washing process also creates a mechanically very harsh environment, the flexibility of the yarn had to be high.

### 2.3. Materials and Methods for the Electronics’ Integration

The yarns were fully woven into the narrow fabric and all the tested versions had additional protection (see Table 1 “Coating” column). Thus, the narrow fabrics were laser cut with a CO_2_ laser in designated positions to create openings for the electronic interconnections (Figure 4c).

Before connecting the electronics to the conductive yarn, they had to be aligned with the opening and fixed on the fabric. Thus, a mechanical connection between the narrow fabric and the PCBs was achieved using an NCE (non-conductive epoxy) that was cured through the textile with a UV light. A small pitch prohibited the creation of an interconnection between the yarns and the electronics with conductive glue due to the low viscosity of the epoxy which created shorts. Therefore, soldering with Class 3 SAC305 solder paste with a melting point of around 220 °C was carried out (Figure 4c). Due to the larger solder powder size, less residue was left between the solder pads on the board.

Before full encapsulation of the sensor boards, they were coated for additional protection with a film coating (ELPEGUARD^®^ Twin-Cure^®^ DSL 1600 E-FLZ, Lackwerke Peters GmbH & Co. KG, Kempen, Germany) to enhance the reliability and longevity of electronic assemblies. The film coating was cured with UV light. The encapsulation of the sensor boards on the textile was performed with a casting compound through low-pressure injection moulding. A two-component polyurethane-based casting compound (ELPEGUARD^®^ VU 4443/92 WR-NV—Lackwerke Peters GmbH & Co. KG, Kempen, Germany) was used and different moulds were tested to reach the optimal protection and transition from rigid electronics to highly flexible textile ribbons.

**Figure 5:**
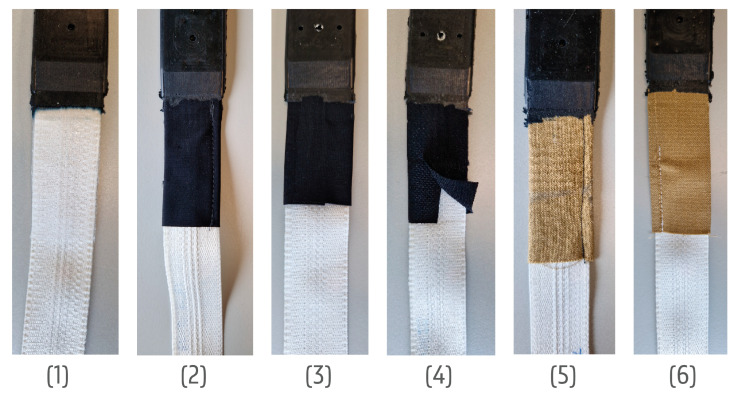
Cast samples (with yarn nr 5) with 6 additional transition materials.

**Figure 6:**
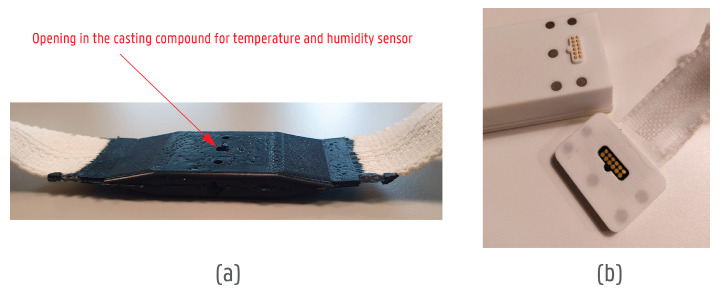
Overview of encapsulation methods for electronic components. (**a**) The encapsulated sensor is on the narrow fabric with an opening in the encapsulation to keep the temperature and humidity sensor open. (**b**) The connector PCB board is encapsulated in a 3D-printed casing together with the CPU box.

The polyester-based ribbon had great softness and flexibility. Thus, between the ribbon and the encapsulation, the casting compound created a more rigid transition. Hence, smooth and protective transitions between the casting compound and textile were needed. In comparison to a textile, the casting compound’s flexibility was much lower, which caused a higher degree of yarn and textile flexing at transition points. Thus, creating a smoother rigid-to-flexible transition was important. Figure 5 shows six additional transition materials tested to preserve the yarns’ durability, and the flexing radius of the yarns was lowered. To gather accurate data for the firefighters, the temperature and humidity sensor was left open using silicone that was later removed (Figure 6a).

The transition materials were:

(1) A 100 µm TPU;
(2) A 213 g/m^2^ knitted fabric sewn around the ribbon;
(3) A 213 g/m^2^ knitted fabric laminated with 100 µm TPU looped around the ribbon;
(4) A 213 g/m^2^ knitted fabric laminated to the narrow fabric halfway with 100 µm TPU;
(5) Meta-aramid fabric sewn around the ribbon;
(6) Meta-aramid fabric laminated with 100 µm TPU looped around the ribbon; laminated halfway and sewn from the side.

All additional versions of the transition material were added to the ribbon before the application of the casting compound

### 2.4. Materials and Methods of the Different System Parts’ Connection 

The connector PCB board was encapsulated using a 3D-printed casing (Figure 6b). The casing included an opening for the CPU box connection contact pads and magnets. Connector boards connected to the narrow fabrics were glued to the 3D-printed box, and silicone paste (RS PRO White Silicone Sealant Paste) was added at the transition between box and fabric to create a soft and smooth transition. 

The CPU box, including the CPU board and battery, was 3D printed from PA12 material to protect the contents from possible high temperatures and, thus, possible heat damage to the end user. The CPU board connection point to the system had pogo pins that could connect to the contact pads on the connector board on the narrow fabric. Additionally, the CPU box was equipped with corresponding magnets that connected to the 3D-printed casing around the connector board, ensuring a tight connection between the pogo pins and the connector.

### 2.5. Methods of the Final System’s Integration into a Product 

The final system consisted of 2 encapsulated sensor boards—each on one shoulder area—and one connector board ending in the chest Napoleon pocket (see more in Figure 2). The CPU box was removable via the magnetic connection (Figure 6b). The narrow fabric carrying the electronics was not stitched fully into the product. Instead, several points along a narrow fabric edge were stitched to complete the final integration, making the system easier to disassemble from the product when necessary. 

Table 2 summarizes the materials and methods used in this study to make samples.

### 2.6. Reliability Testing Methods

#### 2.6.1. Washing Tests

After sample preparation, the integrated narrow fabrics were washed according to the ISO 6330-2012 standard at 60 degrees Celsius [29]. The washing machine used was Electrolux W465H, Stockholm, Sweden. The goal was to reach a minimum of 25 washing cycles with the following parameters:

- Without electrical connection failures in the yarns and connection between yarns and electronics;
- Without losing conductivity in the yarns;
- Without breaking the sensor board, connections to components, and/or components themselves.

**Figure 7:**
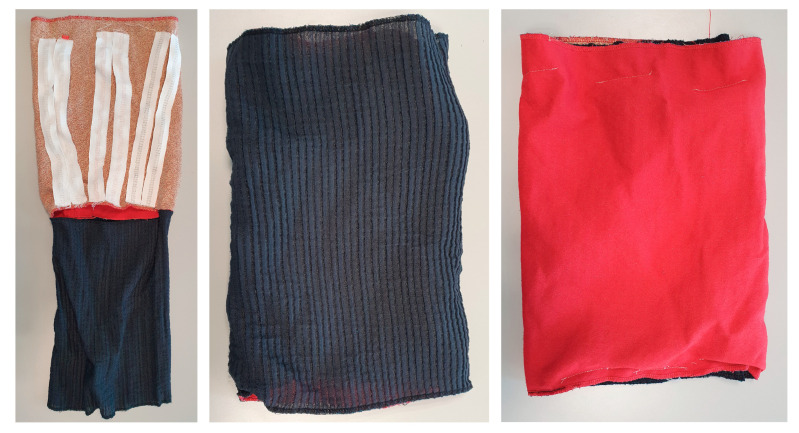
The washing tests for narrow fabrics and electronics integration were conducted in sleeve mock-ups.

Samples were sewn into sleeve mock-ups for washing tests to mimic the final product’s needs (Figure 7). When smaller-scale sample testing was finished, the full system was tested similarly by sewing it into a full product.

#### 2.6.2. Bending Tests

**Figure 8:**
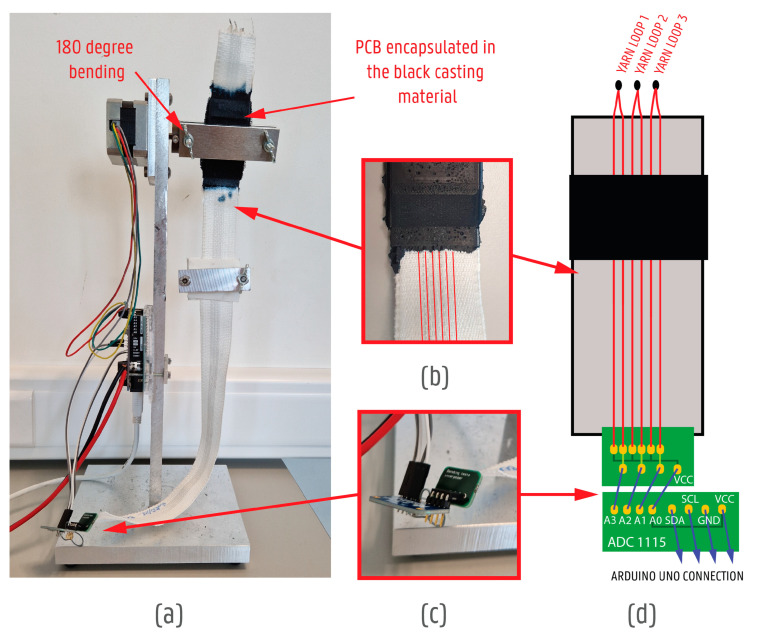
Set-up for testing with motor in the top left, where 180-degree rotation also occurs. (**a**) Machine set-up highlighting where the 180-degree bending takes place. (**b**) A close-up photo of the soft–rigid transition area. (**c**) Close-up photo of the connection between the ADC and the sample. (**d**) Technical drawing of the sample set-up for the bending test.

Additionally, the flex life of the full system, and more specifically the conductive yarns, was tested through bending tests. While washing reliability testing is important, it is also time consuming. During washing tests, it is difficult to pinpoint the time when the yarns begin to break and find methods to avoid it. Thus, a bending test was developed using a stepper motor (Nema 17 motor with Motorshield V2, Adafruit Industries, LLC, New York, NY, USA), Adafruit ADC1115 analogue-to-digital converter (Adafruit Industries, LLC, New York, NY, USA), and Arduino UNO microcontroller (Arduino, Ivrea, Italy). A sample with extra weight was attached between the (black) encapsulated part and the motor and flexed 180 degrees (Figure 8).

Data read-outs included the voltage of the yarns (V); the number of cycles performed; and time (s). Through the bending tests, it was also possible to compare the additional transition methods more accurately by defining the exact yarn degradation in the narrow fabric.

Seven samples with encapsulated electronics (Figure 5) were prepared for a case study:

- Sample 0—Narrow fabric with yarn nr 5;
- Sample 1—Narrow fabric with yarn nr 5 and an additional 100 µm TPU transition where the black casting material meets the textile ribbon;
- Sample 2—Narrow fabric with yarn nr 5 and an additional 213 g/m^2^ knitted fabric looped around the ribbon by sewing where the black casting material meets the textile ribbon;
- Sample 3—Narrow fabric with yarn nr 5 and additional fully laminated 213 g/m^2^ knitted fabric looped around the ribbon by lamination where the black casting material meets the textile ribbon;
- Sample 4—Narrow fabric with yarn nr 5 and an additional 213 g/m^2^ knitted fabric (not laminated itself) looped around the ribbon by lamination where the black casting material meets the textile ribbon;
- Sample 5—Narrow fabric with yarn nr 5 and additional woven aramid fabric looped around the ribbon by sewing where the black casting material meets the textile ribbon;
- Sample 6—Narrow fabric with yarn nr 5 and additional woven aramid fabric (not laminated itself) looped around the ribbon by lamination where the black casting material meets the textile ribbon;
- Sample 7—Narrow fabric with yarn nr 7.

The six yarns in each ribbon were looped into 3 pairs—from one end the power was given in and from the other end the voltage was read-out together with the power input and stepper motor connection. The original voltage (without any degradation to the yarns) was 4.94 V. When the voltage dropped below 3.8 V, the yarn loop was considered to have failed.

#### 2.6.3. Laboratory and Field Tests for Final Integration

All the materials and composite prototypes were first tested in controlled laboratory tests using heat-sensing [30,31] manikins and making sure all the materials and integration methods adhere to EN 469:2005 standard [32]. Throughout the development process, the systems were used with jackets during controlled firefighters’ training exercises. Controlled exercises used modified shipping containers to simulate various firefighting scenarios. Moreover, fire props were installed inside the containers to simulate different types of fires, wood, or flammable liquids, also illustrated by Kaczke (2017) [33]. The data from the users were gathered through discussions and free-form conversations.

During final testing, 9 types of PPE were used for 6 months in real-life conditions. Thus, firefighters used the jackets regularly for six months in their daily work. The aim was to see how these jackets could be integrated into current firefighters’ daily regimes and interventions. During the testing period, firefighters used the jackets in their daily work. Among these risks were extreme temperatures, exposure to hazardous materials, wear and tear from rigorous activities, and the need to fully clean and maintain them regularly. The final data from the users after 6 months of testing were collected through a free-form survey covering (1) electronics functionality and ease-of-use; (2) data collection and usefulness; (3) general durability of parts.

## 3. Results

### 3.1. Reliability Testing

Different features of the narrow fabric system were observed during reliability testing:

1. The conductive yarns: material, properties, and reliability;
2. The encapsulation and the transition from the rigid to the soft parts: encapsulation of electronics within the textile and the transition between the electronics, encapsulation material, and the narrow fabric;
3. The connection method CPU and battery box.

Two durability tests were conducted: washing tests based on ISO 6330-2012 standard at 60 °C and with a type 3 detergent (according to the standard) and bending tests with a tabletop testing machine.

#### 3.1.1. Conductive Yarns

**Figure 9:**
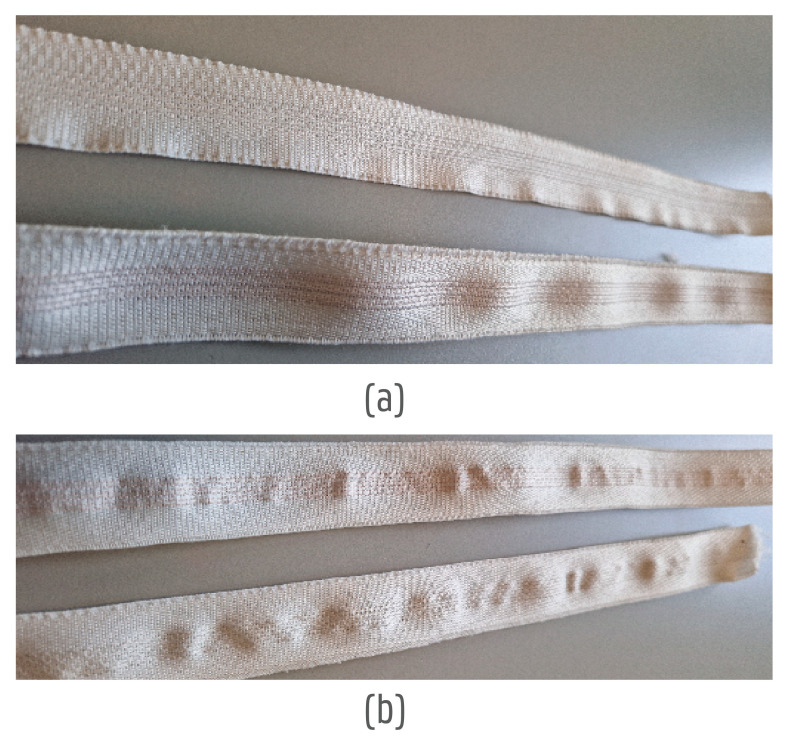
Overview of yarns in the narrow fabric focusing on the yarn tension and drapability. (**a**) Yarns nr 5 (top) and 7 (bottom). (**b**) Yarns nr 1 (top) and 2 (bottom).

The results from the washing tests without electronics and casting compounds can be seen in Table 3. Here, the first set of results is shown in the second column. The most successful yarns were multifilament-based yarns with an additional individual coating (yarns nr 5, 6, 7). The steel composition of yarn nr 7 extended its flex life compared to yarn nr 5 and allowed it to withstand 25 washing cycles. The yarn rigidity, however, was much higher than expected, making it difficult to connect (flat) electronic components (Figure 9). The monofilament yarns nr 1 and 2 broke after five washing cycles due to a low flex life. In addition, these yarns resembled common wires the most in both feel and appearance (Figure 9b). As a result, their drapability was low and they were rigid and the textile was under a larger amount of tension. Yarns nr 3 and 4 had silver bases and were already brittle due to the laser cutting carried out to make the openings. Nevertheless, these yarns drew the most attention for their textile-like touch, feel, and draping qualities.

The second round of results (displayed in the third column) included samples that were most successful in the first round and were easy to handle during manufacturing. While both yarns nr 5 and 7 had their difficulties, they showed the least complications during integration into the narrow fabric and could be processed efficiently. Thus, only yarns 5 and 7 were tested further together with electronics and a casting compound, as yarn samples 1–4 started failing very early in the first tests. Yarn 6 was also not included due to the complicated processing and manufacturing of the narrow fabric since the PA coating was non-slippery and did not run through the weaving machines smoothly. Table 3 shows that yarn 7 had the highest durability reaching 35 washing cycles at 60 degrees without any failures. After the 40th washing cycle, issues occurred, such as the loss of contact with one of the sensors or the inability to collect data in a stable manner.

#### 3.1.2. Encapsulation and the Transition between Rigid and Soft Parts

Samples with yarn nr 7 and the electronics casting compound had no functionality issues after 25 washing cycles. Yet, the drapability and yarn tension were considerably better compared to samples with yarn nr 5. However, samples with yarn 5 and the casting material started to break after around 5–10 washing cycles. Thus, it was seen that the reliability of samples with yarn nr 5 without an additional transition from the casting compound to the ribbon was not sufficient.

**Figure 10:**
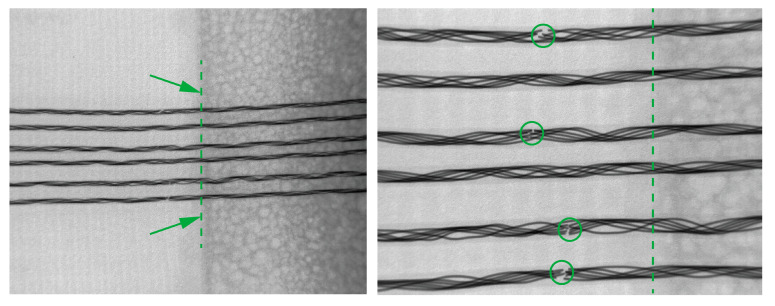
Photos of the broken yarn under x-ray. The yarn’s diameter is 0.6 mm and the pitch between the yarns is 1.27 mm. The arrows and circled areas highlight the breaking points and dashed lines highlight the soft-rigid transition areas.

From the X-ray analysis of the failed sample, it was seen that the main failure point was the transition area from the more rigid casting compound to the narrow fabric, as mentioned before. The conductive yarns in the narrow fabric were not durable enough for such intensive flexing during the tumbling in the washing process, resulting in breakage (Figure 10). Thus, the third round of results included samples with additional transitions around the encapsulation and textile area (Table 3, last column). All the transitions, excluding nr 2 and 5 (Figure 5), increased the washing durability from five washing cycles to thirty washing cycles.

#### 3.1.3. The Connection Method of the CPU and Battery Box

Based on users’ feedback and tests, the connection to the CPU was designed to be smoother and easier. The CPU together with a rechargeable battery was placed in a high-temperature resistant casing with an opening to connect the system. The connection was made with pogo pins touching an interposer PCB connected to the end of the narrow fabric (Figure 6b). The connection was secured with samarium cobalt magnets to ensure no degradation due to the possible high temperatures applied to the system.

### 3.2. Sustainability

Environmental sustainability could be viewed from two aspects: (a) the full product or (b) the e-textile system.

The full product’s sustainability was assessed by examining the ease of reusing parts if or when the system stops working. Specifically, the system’s design considered whether it could be easily removed from the product, repaired, and/or replaced. In the final version, the system was sewn into the jacket’s layers and made accessible by opening a zipper at the bottom hem. Sewing was carried out in specific areas, e.g., every 20 cm, with small local stitches, facilitating easier removal or replacement of the system.

The e-textile system itself was designed and tested for maximum durability. A multifilament yarn was used to ensure higher reliability; even if some filaments broke, others maintained their connection. This design choice contributes to the system’s sustainability due to its long lifespan. However, a major challenge identified is the full encapsulation of electronic parts, which prevents local repairs. If one component, such as a sensor board, fails, the entire system must be replaced, impacting the overall sustainability negatively.

The concept of “full product sustainability” was based on these assessments, ensuring that the product’s components could be reused or replaced efficiently, thereby reducing waste and resource consumption. This holistic approach underscores the need for further development in the repairability of encapsulated electronic parts to enhance the system’s overall sustainability. For environmental sustainability to be fully addressed, the lifecycle analysis (LCA) should still be performed, focusing on the materials used and the sustainability of their design.

### 3.3. User Acceptance

The user acceptance was tested and defined through discussions held throughout the development process. The data from the users were gathered in free-form discussions and analyzed together with all the partners in meetings after every development phase/new prototype version. The data collection was always carried out during or right after testing. For the 6-month trial, the user data were collected at the end in a free-form discussion.

After real use for 6 months, it is clear that different points still need to be changed to make the system acceptable for real use. Therefore, a deeper understanding of user acceptance is necessary. For the electronics side, it was highlighted that recharging the battery of the system should be carried out without extra handling of the system. Therefore, the users preferred not to remove the CPU box during charging, and thus it should be carried out wirelessly. Additionally, shorter charging times and larger battery capacities were emphasized. For the data collection side, the methods for archiving and evaluating data should also be improved. It was also emphasized that communication for data transport and software support should be easier. In general, methods for archiving and evaluating data should also be improved. Enhanced communication for data transport and software support was emphasized to simplify user interactions.

However, cleaning of the jackets, which is performed by an external service provider for the firefighter brigades, was carried out with no difficulty or issues. Thus, it was seen that the general durability of the jacket was acceptable. Nevertheless, it was seen that the period of 6 months is still too short to obtain OELs (Occupational Exposure Limits) for the washing conditions. Therefore, the cleaning period should be extended to ensure that the jackets are properly washed and free from contaminants. Furthermore, the specific process followed for cleaning could be monitored regularly to ensure the quality is upheld.

## 4. Discussion

This work focused on two key aspects: (e-textile system) engineering and evaluation. The engineering features focused on the choice of materials used for the system (textile and electronic), the methods used for the (electronic and functional textile) parts’ connections and integration, and the methods used for the final system’s integration. All of these aspects were tested for reliability through washing and/or bending tests. Additionally, sustainability and user acceptance were considered.

Narrow fabric-based e-textiles, like ribbons and tapes, offer versatile integration options. They can be stitched, or incorporated into textiles, making them ideal for textile-based wearable applications. Moreover, the manufacturing of the final product is significantly easier since the textile-based electronic system can be separately made and sewn into the final product. Current research suggests that conductive yarns’ integration into narrow fabrics has some downsides, and some aspects must be examined before using them as an interconnect bus system. Furthermore, high-stress areas were found around transition points between textiles and encapsulating materials for electronics protection. These had to be mitigated and transitions had to be made smoother. Thus, the following points should be considered before using this technology for an integrated e-textile bus-system:

1. The used conductive yarn’s thickness and rigidity;
2. The availability of coatings for additional protection of the conductive yarn used;
3. The used conductive yarn’s electrical resistance;
4. The compatibility of the conducive yarn’s material with electronics, e.g., whether soldering is possible;
5. The possibility of weaving conductive yarns with a specific pitch;
6. How stable are the yarns and the pitch between them after durability tests, e.g., washing, and will shorts appear after some handling.

Moreover, environmental sustainability was considered from two aspects: (a) the full product or (b) the e-textile system. A functional narrow fabric system could easily be removed from the full product. The system parts cannot be repaired due to the full encapsulation of all electronic parts. The full encapsulation of electronic parts in the system prevents any possibility of repair, which can be problematic from an environmental standpoint. This means that if any component of the e-textile system fails, the entire system would need to be discarded and replaced, contributing to electronic waste. However, the e-textile system was designed and tested to be as durable as possible, allowing it to last for longer periods (at least 35 washing cycles at 60 degrees Celsius and bending 180 degrees in high-stress areas 100,000 times).

One alternative approach to repairing e-textile systems without full encapsulation could be the use of modular components. The system could be designed with interchangeable parts so that only the faulty component would need to be replaced instead of the entire system. However, that can lead to reliability issues during use, such as unreliable connections between modular components.

The user acceptance was tested and defined by discussions throughout the development process. After real use for 6 months, different points still needed to be changed to make the system acceptable for real use. Therefore, a deeper understanding of user acceptance is necessary. For example, it was highlighted that powering the system should be carried out without extra handling of the system. Therefore, the users preferred to keep the CPU box inside the garment during charging and that charging would be then carried out wirelessly. Additionally, shorter charging times and larger battery capacities were emphasized.

Methods for archiving and evaluating data should also be improved. It was also emphasized that communication for data transport and software support should be easier. However, cleaning of the jackets, which is provided by external service providers for the firefighter brigades, was carried out with no difficulty or issues. Users’ feedback could be incorporated into the system’s development and usability testing to identify and address any remaining problems. Additionally, implementing features such as automatic system updates, intuitive data management tools, and simplified communication channels could enhance the overall user experience and make the system more user-friendly. Considering a cloud-based storage system that allows easy access and retrieval of data could improve the archiving and evaluation of data. In addition, users could be provided with insights and visualizations of the collected data using data analytics tools. Finally, conducting regular user surveys and feedback sessions can provide valuable information on how to further improve the data archiving and evaluation processes.

## 5. Conclusions

This work introduces several novel aspects that advance the field of e-textile systems, particularly in the context of PPE. The integration of narrow fabric-based e-textiles into PPE is a key innovation, offering versatile and practical solutions for embedding electronic functionalities into garments. This method allowed for the separate manufacturing of the textile-based electronic system, which could then be sewn into the final product, simplifying production processes. Although the current fully encapsulated design prevents repairability, the study suggests exploring the use of modular components to reduce electronic waste, a significant environmental concern.

The study highlighted key considerations in material selection, integration methods, reliability, sustainability, and user experience. Through such collaborative efforts, the potential of e-textiles to enhance safety and well-being in various fields, particularly in PPE, can be fully realized. Moreover, the study addressed the critical issue of the durability of e-textiles by conducting extensive reliability tests, such as washing and bending tests. The findings highlighted potential weaknesses at the transition points between the textiles and encapsulating materials for electronics, proposing solutions to mitigate these stress points and ensure smoother transitions. In general, this enhanced the overall robustness of the e-textile system and eliminated materials that were not durable enough for current application.

Finally, the study involved continuous user acceptance discussions throughout the development process and real-world testing over six months, identifying practical needs and wants of the end-users. The aims of the user-centred approach was to ensure that the final product meets the practical demands of end-users, enhancing its likelihood of adoption.

Future research directions for improving e-textile reliability and sustainability could include exploring more encapsulation material options that can, for example, be fully degraded or separated from the electronics. Additionally, studying user feedback and incorporating it into the design process can further enhance the overall user experience and ensure that the e-textile system meets the specific needs of the end-users.

## 6. Patents

Title: Methods for protecting conductive yarns at the rigid-soft transition between electronics and fabrics in etextiles—EP 22211836.6 filed on 6 December 2022.

## Figures and Tables

**Table 1:** Overview of conductive yarns tested to reach the best durability, integration ease and user acceptance. Yarn suppliers have not been disclosed due to confidentiality.

Nr	Materials	Filaments	Coating	Resistance (Ω/m)
1	Cu-based	Mono-1	Polyurethane (TW-C)	0.52–0.57
2	Ag/Cu-based	Mono-1	Polyester (TW-H)	0.13–0.14
3	Polyamide core, Ag-based	Multi-34	TPU	50 ± 10
4	Polyamide core, Ag-based	Multi-36	PVC	5 ± 5
5	Polyester core, Ni/Cu-based	Multi-6	PTFE	0.8
6	Polyester core, Cu/Sn-based	Multi-6	Polyamide (PA12)	1.04
7	No core, Cu/steel-based	Multi-14	PTFE	0.99

**Table 2:** Overview of materials and methods used to integrate electronics to narrow fabric.

Action	Material/Method
Narrow fabric material choice and characteristics	Polyester material, width 26.4 mm, pitch between the yarns 1.27 mm
Yarn selection	Seven different yarns tested
Yarn/casting compound additional transition	Six different material combinations tested
Laser ablation	CO_2_ laser
Electronics’ connection to the narrow fabric	With NCE (Loctite AA 3526, Henkel, Brussels, Belgium)
Electrical interconnection to the conductive yarns	With class 3 solder paste (DP 5505 SAC 305, Interflux, Ghent, Belgium)
Electronics’ coating	Film coating (ELPEGUARD^®^ Twin-Cure^®^ DSL 1600 E-FLZ, Lackwerke Peters GmbH & Co. KG, Kempen, Germany)
Electronics’ encapsulation material and method	Casting compound injection-moulded around the electronics (Elepecast VU 4443/92 WR-NV, Lackwerke Peters GmbH & Co. KG, Kempen, Germany)
Connection to the CPU/battery box	With magnets and pogo pins

**Table 3:** Overview of yarns’ reliability to washing tests.

Yarn nr	Washing Durability without Electronics and Casting Compound (Washing Cycles)	Washing Durability with Electronics and Casting Compound (Washing Cycles)	Washing Durability with Electronics and Casting Compound with Additional Transition Materials (Washing Cycles)
1	5	N/A	N/A
2	5	N/A	N/A
3	0	N/A	N/A
4	2	N/A	N/A
5	25	5	35
6	25	N/A	N/A
7	25	25	35

N/A: not applied.

## Data Availability

Data are contained within the article.
